# Ureteric Injuries after Hysterectomy in a Tertiary Care Center of Nepal: A Descriptive Cross-sectional Study

**DOI:** 10.31729/jnma.6684

**Published:** 2021-07-31

**Authors:** Ratna Adhikari Khatri, Arju Chand, Sumana Thapa, Shailaja Khadka, Manish Thapa

**Affiliations:** 1 Department of Obstetrics and Gynecology, Shree Birendra Hospital, Chhauni, Kathmandu, Nepal; 2 Department of Radiology, Shree Birendra Hospital, Chhauni, Kathmandu, Nepal

**Keywords:** *hysterectomy*, *iatrogenic*, *injury*, *ureter*

## Abstract

**Introduction:**

Pelvic surgery is the most common cause of iatrogenic ureteral injury. The incidence of ureteric injuries varies between skilled and inexperienced surgeons. The study aims to determine the prevalence of ureteric injuries sustained during hysterectomy in a tertiary care center of Nepal.

**Methods:**

A descriptive cross-sectional study involving the women attending the gynecological outpatient department of a tertiary care center of Nepal, for various benign and malignant conditions and later on underwent hysterectomy from June 2019 to June 2020 was done after obtaining ethical clearence from the Institutional Review Committee (Reference No. 245). Convenient sampling method was used. The data were entered in Excel and analyzed using Statistical Package for Social Sciences version 17. Point estimate at 95% Confidence Interval was calculated along with frequency and proportion for binary data.

**Results:**

Altogether, 1 (0.63%) (0.55-0.71 at 95% Confidence Interval) out of 159 patients sustained the ureteric injury during hysterectomy in a tertiary care center of Nepal. The injury was seen during the exploratory laparotomy for adnexal mass. The injury was recognized intraoperatively and was repaired with double J stenting. A total of 159 patients were enrolled in the study that had undergone hysterectomy over one year for various benign and malignant conditions. Out of which 21 (13.2%) had undergone surgeries for malignant conditions and 138 (86.79%) for benign conditions.

**Conclusions:**

Iatrogenic ureteric is still a major cause of harm and concern in hysterectomy. Patients with ureteric injury should be evaluated and intervened at the earliest.

## INTRODUCTION

The incidence of ureteric injury is reported to be 0.5 to 1.0% in surgery of benign diseases^1–3^ and 5 to 30% in surgery of malignant diseases like radical hysterectomy.^2^ Ureteric injury is reported more when patients have associated factors like endometriosis, pelvic infection, huge pelvic masses, previous abdominopelvic surgery, and radiation therapy.^4,5–6^ Ligation and transaction injuries are the most common type, and other forms are thermal injury, kinking, de-vascularization, partial/complete transaction, and perforation.^7–8^

Iatrogenic ureteral injury is a serious complication of gynecologic surgery. The idenfication of these injuries needs to be done on time which will allow the immediate repair of these injuries, reduce morbidity, and decrease the chances of the medico-legal outcome. To our knowledge, such study hasn't been conducted in any hospitals of Nepal till date.

The study aims to find out the prevalence of ureteric injuries sustained during hysterectomy in a tertiary care center of Nepal.

## METHODS

This descriptive cross-sectional study was conducted in Shree Birendra Hospital (SBH), a tertiary level hospital in Kathmandu, Nepal. The Institutional Review Committee of the Nepalese Army Institute of Health Sciences approved the study (Reference No. 245), and informed written consent was obtained from all the patients before enrolment. This study included women visiting the Gynecology outpatient department of SBH with benign and malignant indications to undergo an elective hysterectomy in one year between June 2019 to June 2020. Laparoscopic hysterectomy were excluded from the study. The sample size was calculated as follows:

n = Z^2^ × p × q / e^2^

  = (1.96)^2^ × 0.5 × (1-0.5) / (0.09)^2^

  = 119

Where,

n = minimum required sample size
Z = 1.96 at 95% Confidence Interval (CI)
p = prevalence taken as 50% for maximum sample size
q = 1-p
e = margin of error, 9%

The calculated sample size was calculated to be 119. Adding 10% as a non-response rate, the minimum required sample size was 131. We took data from 159 patients. Convenient sampling method was used.

The data was collected using a semi-structured questionnaire consisting of variables such as age at the time of surgery, indication for surgery, type of surgery performed, type and time when the injury was identified, method of repair, and outcome of the repair. The data was obtained from the patient by interviewing, from case files, operating theatre details, surgical and gynecological ward registries. Visualization of ureteric peristalsis and pooling of urine in the operating field was the initial intraoperative method to detect injury during the procedure. The injury identified at the table was repaired during the primary surgery by the attending gynecologist and urosurgeon.

The data were entered in Excel and analyzed using Statistical Package for Social Sciences (SPSS) version 17.

## RESULTS

Altogether, 1 (0.63%) (0.55-0.71 at 95% Confidence Interval) out of 159 patients sustained the ureteric injury after hysterectomy in our study. The injury was seen during the exploratory laparotomy for adnexal mass.

**Figure 1:**
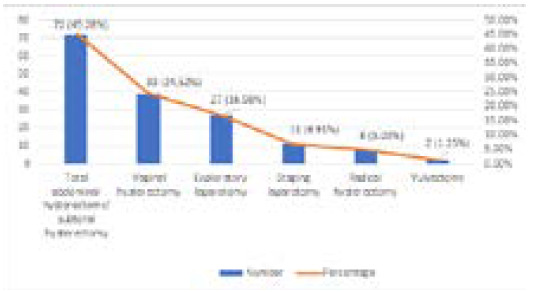
Types of surgery conducted during the study period.

There were 1 59 patients in the study group. The average age of the patients was 48 years ranging from 32 years to 73 years. The most common surgery was total abdominal hysterectomy 72 (45.28%), followed by vaginal hysterectomy 39 (24.52%). About 27 (16.98%) patients underwent exploratory laparotomy where one case was met with a ureteric injury (Figure 1).

Altogether, 1 (0.63%) patient sustained the ureteric injury.

**Figure 2:**
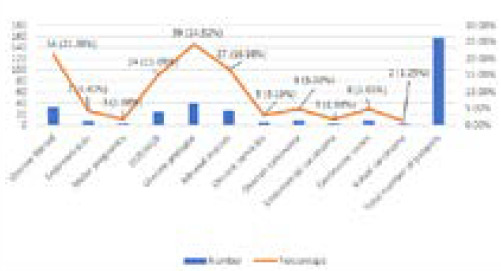
Pathology for which operations were performed.

Twenty-one (13.2%) had undergone elective hysterectomy surgeries for malignant conditions and 138 (86.79%) for benign conditions. The group of benign conditions included uterine prolapse 39 (24.52%), uterine fibroids 34 (21.38%), benign adnexal masses 27 (16.98%), menstrual disorders 24 (15.09%), and endometriosis 7 (4.40%). The group of malignant conditions was limited to ovarian cancer 8 (5.03%), cervical cancer 8 (5.03%), endometrial cancer 3 (1.88%), molar pregnancy 3 (1.88%), vulval cancer 2 (1.25%). The study distinguished between the open procedures followed for benign conditions and malignant conditions. Benign conditions had undergone total abdominal hysterectomy, subtotal hysterectomy, vaginal hysterectomy, and exploratory laparotomy. Among malignant conditions, the surgical operations conducted were staging laparotomy, radical hysterectomy, and vulvectomy (Figure 2).

## DISCUSSION

Iatrogenic injury to the ureter is the most common complication of abdominopelvic surgery, ranging from less than 1 to 10 percent of procedures, depending upon the complexity of the procedure.^9-11^ In this study, the iatrogenic ureteric injury was reported in 0.63% of all types of hysterectomy, a figure which is comparable with other literature.^10,11^ However, the figure of 0.63% towards the lower side of the studies in the literature may probably be that the trained gynecologists perform all hysterectomies in our institution and another factor may be the laparoscopic hysterectomy is excluded from the study. Gynecologic laparoscopic procedures account for more than half of the injuries, and the most common location is the lower ureter.^5,12^ The injury or fistula may become apparent either immediately or much more commonly, in a delayed fashion several days to weeks after surgery. However, a high index of suspicion with symptoms such as flank pain and fever may suggest ureteric injury after pelvic surgery. Complete transaction of the ureter causes immediate leakage of urine within first 24-48 hours of surgery, while ligation and thermal injury present later following tissue necrosis.

The ureteric injury sustained in this study was following exploratory laparotomy with hysterectomy done for adnexal mass in 73 years old patient. The injury was identified intraoperatively by urinary extravasations into the retroperitoneal space. Similar to the study conducted by Patil SB, et al. open hysterectomy for benign diseases was the cause of ureteric injury.^5^ The time of recognition of injury is crucial. Intraoperative recognition of ureteric injury is very low ranges from 11-33%. If they are identified pre-operatively, they can be repaired quite easily. Otherwise, if these injuries are identified late, patients do present with anuria, renal failure, and urinary fistula. In Khizar Hayat, et al. study, 28.56% patients' injury was identified at the table and managed accordingly. A similar review conducted in eastern Nigeria found ligation and transaction the most common form of injury.^4,7^

The risk factors for iatrogenic injury include nature and indication of the abdominal or pelvic surgery, patient-related factors such as pelvic adhesions from previous surgeries, history of pelvic radiation, enlarged uterus, pelvic malignancy, pelvic endometriosis, and anatomical abnormalities.^9,13,14^ In the present study, the cause of injury was pelvic adhesion with the retroperitoneal adnexal mass and found to have inadvertent ureter.

The experience of the operating surgeon may also be an important risk factor.^15-6^ Keeping this in mind, the hysterectomies in our institution are all conducted by experienced surgeons. This may be why we had a smaller number of iatrogenic ureteric or bladder injuries and an injury identified intraoperatively.

Similar to our study, Blackwell RH, et al. reported 81 percent of those who underwent hysterectomy for benign indications. The ureteral injury occurred in less than 1 percent of patients (0.78%) and was unrecognized in 62 percent of cases.^17^ While there was no significantly increased risk of acute renal failure or death for unrecognized ureteral injuries compared with no injuries, the unrecognized injury was associated with a nearly 24-fold increased risk of acute renal failure and 40 percent increased odds for death.^1^

The patient with ureteric injury should be evaluated and intervened at the earliest within two weeks after hysterectomy because it has higher chances of success with endourological procedures, obviating the need for open surgery.^5^ Thus, to detect any unrecognized injuries, apart from this study, all patients were reviewed and followed up with clinical evaluation, urine routine examination, and ultrasound abdominopelvic in 2 weeks and a month later to detect the problem at the earliest and tackle it to prevent morbidity and mortality.

As this study is conducted in one institution and confined to one population, the findings cannot be generalized. More extensive studies need to be undertaken related to this subject for better analysis.

## CONCLUSIONS

Our study revealed that a simple hysterectomy for a benign disease could be the cause of ureteral injury. Intraoperative evaluation of the ureter during vaginal procedures is more difficult given the limited operating field and anatomic distortion. Cystoscopy is a simple procedure to evaluate a silent injury to the urinary system. The adoption of universal cystoscopy after all hysterectomies is recommended. This will allow the immediate repair of these injuries, reduce morbidity, and decrease the chances of the medico-legal outcome.
